# Clinicopathological and Prognostic Characteristics of Gastric-Type Endocervical Adenocarcinoma: A Nested Case–Control Study

**DOI:** 10.3390/cancers18071168

**Published:** 2026-04-04

**Authors:** Yang Liu, Yundi Hu, Hui Wang, Ling Qiu, Xiaomei Sun, Xuan Yin, Shen Luo, Yue Yin, Qing Cong, Xiang Tao, Yan Ning, Yan Zhao, Haiou Liu, Hua Jiang, Xiaolei Lin, Xin Wu

**Affiliations:** 1 Gynecologic Excellence in Malignancy (GEM-004), Obstetrics & Gynecology Hospital of Fudan University, Shanghai Key Lab of Reproduction and Development, Shanghai Key Lab of Female Reproductive Endocrine Related Diseases, Shanghai 200433, China; liuyang7836@fckyy.org.cn (Y.L.); wanghui10242@fckyy.org.cn (H.W.); 25111250041@m.fudan.edu.cn (L.Q.); sunxiaomei7858@fckyy.org.cn (X.S.); 22211250036@m.fudan.edu.cn (S.L.); yinyue10289@fckyy.org.cn (Y.Y.); jianghua@fudan.edu.cn (H.J.); 2 School of Data Science, Fudan University, Shanghai 200433, China; 23210980108@m.fudan.edu.cn; 3 Department of Radiology, Obstetrics & Gynecology Hospital of Fudan University, Shanghai 200433, China; yinxuan6896@fckyy.org.cn; 4 Cervical Disease Center, Department of Gynecology, Obstetrics & Gynecology Hospital of Fudan University, Shanghai 200433, China; qingcong@fudan.edu.cn; 5 Department of Pathology, Obstetrics & Gynecology Hospital of Fudan University, Shanghai 200433, China; taoxiang1696@fckyy.org.cn (X.T.); ningyan1370@fckyy.org.cn (Y.N.); 6 Obstetrics & Gynecology Hospital of Fudan University, Shanghai Key Lab of Reproduction and Development, Shanghai Key Lab of Female Reproductive Endocrine Related Diseases, Shanghai 200433, China; zhaoyan10251@fckyy.org.cn (Y.Z.); liuhaiou@fudan.edu.cn (H.L.)

**Keywords:** gastric-type endocervical adenocarcinoma, usual endocervical adenocarcinoma, prognosis, adjuvant therapy, clinicopathological features

## Abstract

**Background/Objectives**: Gastric-type endocervical adenocarcinoma (G-EAC) is a rare, aggressive, and HPV-independent subtype of cervical cancer with a poor prognosis. Due to its rarity, existing literature is often limited by small sample sizes, which hinders the development of evidence-based clinical management strategies. This study aims to evaluate the clinicopathological features, prognostic factors, and responses to postoperative adjuvant therapy in a large cohort of G-EAC patients compared with those with usual endocervical adenocarcinoma (UEA). **Methods**: We conducted a nested case–control study within a prospectively maintained surgical cohort at a national referral center in China. The study population included 195 pathologically confirmed G-EAC cases and 765 UEA cases. Patients were followed longitudinally with comprehensive clinical and survival data collection. One-to-one propensity score matching (PSM) was performed to balance demographic, clinical, and treatment variables between the groups. Survival outcomes were compared using Kaplan–Meier analysis, and independent prognostic factors were identified via Cox regression. **Results**: G-EAC patients demonstrated significantly worse survival outcomes than matched UEA patients, with 3-year progression-free survival (PFS) of 66.1% vs. 79.8% (*p* = 0.014) and 3-year overall survival (OS) of 74.9% vs. 84.6% (*p* = 0.033). Parametrial involvement and pelvic lymph node metastasis were identified as independent risk factors for both recurrence and death (*p* < 0.05). Regarding adjuvant treatment, combined radiotherapy and chemotherapy significantly improved survival compared with single-modality treatments (PFS: 65.2% vs. 43.6%; OS: 74.3% vs. 54.5%; *p* < 0.05); however, G-EAC remained less responsive to these therapies than UEA. **Conclusions**: G-EAC exhibits more aggressive clinical behavior and poorer survival outcomes compared to UEA. While combined radiotherapy and chemotherapy offer survival benefits, outcomes remain suboptimal. These findings underscore the urgent need for early detection strategies and the development of more effective targeted therapies for this specific subtype.

## Simple Summary

Gastric-type cervical cancer is a rare and aggressive disease that does not respond well to standard treatments. Because it is so uncommon, many previous studies included only a few patients, making it difficult for doctors to determine the best way to manage the disease. To address this, we studied one of the largest groups of patients to date—195 individuals—and compared their outcomes with those of usual endocervical adenocarcinoma patients. We aimed to identify key risk factors and evaluate how well different therapies work. Our findings show that this cancer type is more likely to recur and lead to poorer survival. By identifying specific risk factors and showing that combined therapy offers better results, this research provides the medical community with much-needed evidence to improve diagnosis and create more personalized treatment plans for these patients.

## 1. Introduction

Cervical cancer is the fourth most common cancer and cause of cancer-related death among women worldwide. In 2022, approximately 660,000 new cases and 350,000 deaths occurred globally [1], with particularly heavy burden in countries with low to medium Human Development Index, where cervical cancer is the second leading cause of cancer incidence and mortality, following breast cancer. While the incidence of cervical squamous cell carcinoma has declined due to human papillomavirus (HPV) vaccination and screening, endocervical adenocarcinoma has risen [2,3,4]. The International Endocervical Adenocarcinoma Criteria and Classification (IECC) and the 5th edition of the WHO Classification divide endocervical adenocarcinomas into HPV-associated and HPV-independent types, with the latter comprising about 20% of cases and showing more aggressive clinical behavior [5,6].

Gastric-type endocervical adenocarcinoma (G-EAC) is the most common HPV-independent subtype, accounting for roughly 2% of all cervical cancers and 10% of endocervical adenocarcinomas [5,7]. First defined by Kojima et al. [8], it was previously known as minimal deviation adenocarcinoma or malignant adenoma. Histologically, it is characterized by glands with abundant clear or eosinophilic cytoplasm and distinct cell borders, resembling pancreaticobiliary adenocarcinoma [9]. Its differentiation ranges from well-formed glands to poorly differentiated clusters or single cells.

The etiology of G-EAC remains uncertain and is unrelated to HPV infection, though some cases occur in patients with Peutz-Jeghers syndrome due to *STK11* mutations [10]. Its pathogenesis may involve a precursor sequence from lobular endocervical glandular hyperplasia to gastric-type adenocarcinoma in situ [11]. Clinically, patients often present with watery discharge or vaginal bleeding. Owing to its HPV-independence and frequent origin in the upper cervix, G-EAC is often missed by cytology-based screening, leading to delayed diagnosis and poor outcomes. Compared with stage-matched usual-type endocervical adenocarcinoma (UEA), G-EAC is associated with worse overall survival and progression-free survival [12].

Despite being recognized as a distinct entity, data on G-EAC remain scarce. Most studies are small retrospective analyses or case reports emphasizing its unique histopathology, aggressive nature, and limited response to chemotherapy [13,14]. Larger studies are required to clarify its biological and clinical characteristics.

This study analyzes the clinicopathological features and prognostic factors of G-EAC diagnosed and treated at our institution between 2018 and 2023. By comparing outcomes with UEA through matched analyses and evaluating the impact of adjuvant therapy, we aim to improve understanding of this rare malignancy and provide evidence for better management strategies. This research is for the GEM-004 Investigation.

## 2. Materials and Methods

### 2.1. Study Design and Population

This study is a single-center, nested case–control study based on a prospectively established surgical cohort at the Obstetrics and Gynecology Hospital of Fudan University. For the present analysis, we retrospectively screened and identified a sub-cohort of patients meeting the inclusion criteria who were enrolled between December 2017 and December 2023. All participants provided written informed consent at the time of cohort enrollment, and the study was approved by the hospital’s Ethics Committee (Approval ID: 2022-51).

### 2.2. Pathological Review and Classification

We specifically focused on patients diagnosed with endocervical adenocarcinoma. It is important to note that all cases in this study were diagnosed postoperatively based on final surgical specimens to ensure diagnostic precision. Adhering to the 5th edition of the WHO Classification, all histological slides were independently reviewed by two senior gynecologic pathologists (Xiang Tao and Yan Ning). Cases with final postoperative pathology confirmed as G-EAC were included in the case group, while UEA served as the control group.

### 2.3. Inclusion and Exclusion Criteria

The inclusion criteria required a confirmed postoperative diagnosis of endocervical adenocarcinoma component.

**Figure 1:**
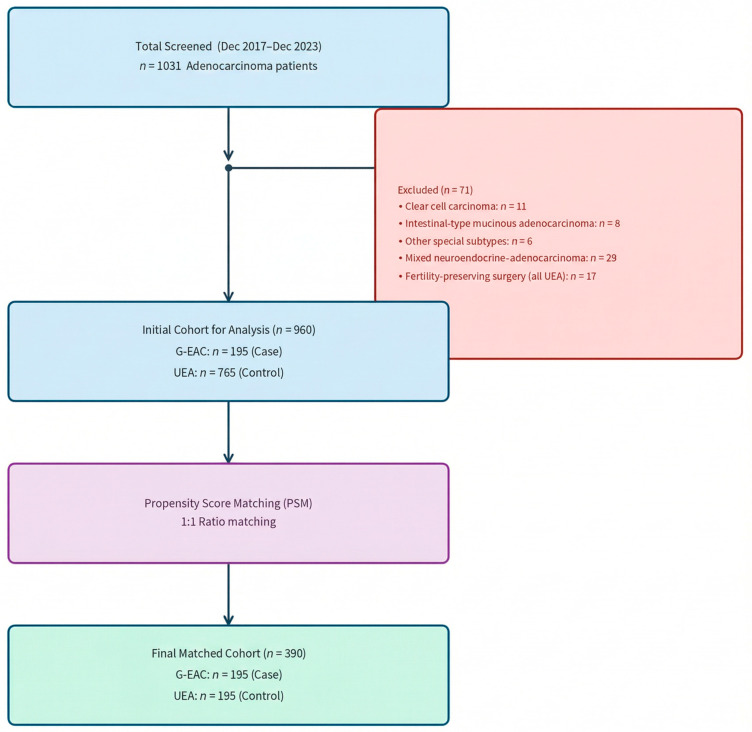
Patient screening and selection flowchart. UEA, usual-type endocervical adenocarcinoma; G-EAC, gastric-type endocervical adenocarcinoma.

Exclusion criteria were: (1) other pathology subtypes (e.g., clear cell carcinoma, mesonephric carcinoma, intestinal-type mucinous adenocarcinoma, signet ring cell carcinoma, invasive stratified mucin-producing carcinoma, mixed neuroendocrine-adenocarcinoma); and (2) patients undergoing fertility-preserving surgery. The exclusion of fertility-preserving cases was intended to maintain a consistent standard of surgical radicality across both groups, thereby eliminating potential survival bias. The detailed screening and selection process is illustrated in the Flowchart (Figure 1).

### 2.4. Data Collection

We collected demographic, clinical, treatment, pathological, tumor marker, and follow-up data. Demographics data included age, ethnicity, marital status. Clinical data included year of diagnosis, BMI, Karnofsky Performance Status (KPS), menstrual and reproductive history, and 2018 FIGO stage. Treatment data included surgical approach and adjuvant therapy. Tumor markers included CA125 and CA199. Pathology data included histological type, tumor size, stromal invasion depth, parametrial or vaginal involvement, margin status, ovarian metastasis, lymphovascular space invasion (LVSI), and lymph node involvement.

Outcome data were obtained through annual telephone follow-ups via the hospital’s cervical cancer follow-up platform, recording disease recurrence (timing, site, treatment), survival status, and date of death. The follow-up ended on 31 December 2024.

Adjuvant therapy decisions were based on the presence of intermediate or high-risk pathological features. Therapies included radiotherapy, chemotherapy, or concurrent chemoradiotherapy, with or without sequential chemotherapy. All patients were staged using the 2018 FIGO classification system [15].

### 2.5. Adjuvant Treatment and Follow-Up

Specifically, patients with high-risk factors (including lymph node metastasis, parametrial involvement, or positive surgical margins) received adjuvant radiotherapy with or without concurrent chemotherapy. For those with intermediate-risk clinicopathological features (considering tumor size, depth of stromal invasion, and lymphovascular space invasion), adjuvant treatment decisions were guided by comprehensive institutional protocols and the multidisciplinary team (MDT) consensus, with reference to the NCCN Guidelines for Cervical Cancer. The specific choice of chemotherapy, radiotherapy, or concurrent chemoradiotherapy followed the standardized care at the time of treatment.

All patients in this cohort were followed up according to a standardized protocol based on the NCCN Guidelines for Cervical Cancer. Patients were generally evaluated every 3 months for the first 2 years, and every 6 months for the subsequent 3 years, and annually thereafter based on patient’s risk of disease recurrence. Consistent imaging (e.g., pelvic MRI or CT) and physical examinations were utilized during these visits, ensuring that the detection interval for recurrence remained comparable across both groups.

### 2.6. Endpoints

The primary endpoints were 3-year overall survival and progression-free survival. Overall survival was defined as time from surgery to death from any cause; censored at last known follow-up for surviving patients. Progression-free survival was defined as time from surgery to first recurrence or death, whichever occurred first; censored at last follow-up if no event occurred. Progression was confirmed through clinical assessment, imaging (MRI, CT, PET/CT), tumor markers, or histopathology from biopsy/reoperation.

### 2.7. Statistical Analysis

Continuous variables were expressed as mean ± standard deviation (SD) or median (interquartile range), and compared using the *t* test or Mann–Whitney U test, as appropriate. Categorical variables were reported as counts (%) and compared using the Chi-square test.

One-to-one propensity score matching (PSM) was performed between gastric-type and usual endocervical adenocarcinoma patients using demographic, clinical, and treatment variables including age, ethnicity, marital status, year of diagnosis, BMI, KPS, menopausal status, parity, FIGO stage, surgical approach, and adjuvant therapy. A greedy nearest-neighbor algorithm with a caliper width of 0.2 of the pooled standard deviation of logit-transformed propensity scores was used [16,17]. Standardized mean difference (SMD) < 0.2 indicated adequate covariate balance.

$$SMD=\frac{{\bar{X}}_{1}-{\bar{X}}_{2}}{\sqrt{\left({sd}_{1}^{2}+{sd}_{2}^{2}\right)/2}}$$

Survival curves were generated using the Kaplan–Meier method, with differences assessed by the log-rank test. In both groups, univariate analysis identified prognostic pathological features (*p* < 0.05), which were entered into multivariate Cox proportional hazards models to determine independent prognostic factors. The Cox model also assessed the effect of adjuvant therapy and its interactions with high-risk pathological factors. A two-sided *p* < 0.05 was considered statistically significant. Analyses were conducted using R (version 4.0.5; R Foundation for Statistical Computing, Vienna, Austria).

In accordance with the journal’s guidelines, we will provide our data for independent analysis by a selected team by the Editorial Team for the purposes of additional data analysis or for the reproducibility of this study in other centers if such is requested.

## 3. Results

A total of 1031 patients diagnosed with endocervical adenocarcinoma who underwent surgical treatment at our institution between December 2017 and December 2023 were initially screened. Following the pre-defined exclusion criteria, 71 patients were excluded: 11 cases of clear cell carcinoma, 8 cases of intestinal-type mucinous adenocarcinoma, 6 cases of other special adenocarcinoma subtypes, 29 cases of mixed neuroendocrine-adenocarcinoma (e.g., small cell neuroendocrine carcinoma combined with invasive adenocarcinoma), and 17 who underwent fertility-preserving surgery (all in the UEA group). Consequently, an initial cohort of 960 patients was eligible for analysis (Figure 1),comprising 195 cases of G-EAC and 765 cases of UEA.

Patients with G-EAC were older (mean age 50.29 vs. 46.76 years, *p* < 0.001) and more often postmenopausal (45.1% vs. 30.3%, *p* < 0.001). They more frequently presented with advanced FIGO stage (≥IIB) (56.5% vs. 12.6%, *p* < 0.001) and had higher rates of abdominal surgery (14.9% vs. 5.6%, *p* < 0.001) and adjuvant therapy (89.7% vs. 41.0%, *p* < 0.001) (Table S1).

In the unmatched population, patients with G-EAC had higher serum CA199 and CA125 levels and differed significantly in pathological features and outcomes compared with usual endocervical adenocarcinoma (Table S1).

To minimize potential confounding bias between the two groups, a 1:1 PSM was performed based on age, ethnicity, marital status, year of diagnosis, BMI, KPS, menopausal status, parity, FIGO stage, surgical approach, and adjuvant therapy. The final matched cohort consisted of 390 patients, with 195 patients in each group (Figure 1). The two groups were well balanced for key covariates (Table S2).

Patients with G-EAC had higher CA199 levels (44.26 vs. 9.73, *p* < 0.001) with no significant difference in CA125. They also exhibited more aggressive features, including ovarian metastasis (15.0% vs. 4.9%, *p* = 0.003), maximum tumor diameter > 4 cm (39.7% vs. 24.2%, *p* = 0.002), deep 1/3 stromal invasion (84.6% vs. 63.1%, *p* < 0.001), parametrial invasion (11.3% vs. 2.6%, *p* = 0.001), positive margins (16.9% vs. 4.1%, *p* < 0.001), and vaginal invasion (44.6% vs. 26.2%, *p* < 0.001). Recurrence was more frequent (27.2% vs. 17.9%, *p* = 0.039), and mortality was higher but not statistically significant (20.5% vs. 12.8%, *p* = 0.057) (Table 1).

**Figure 2:**
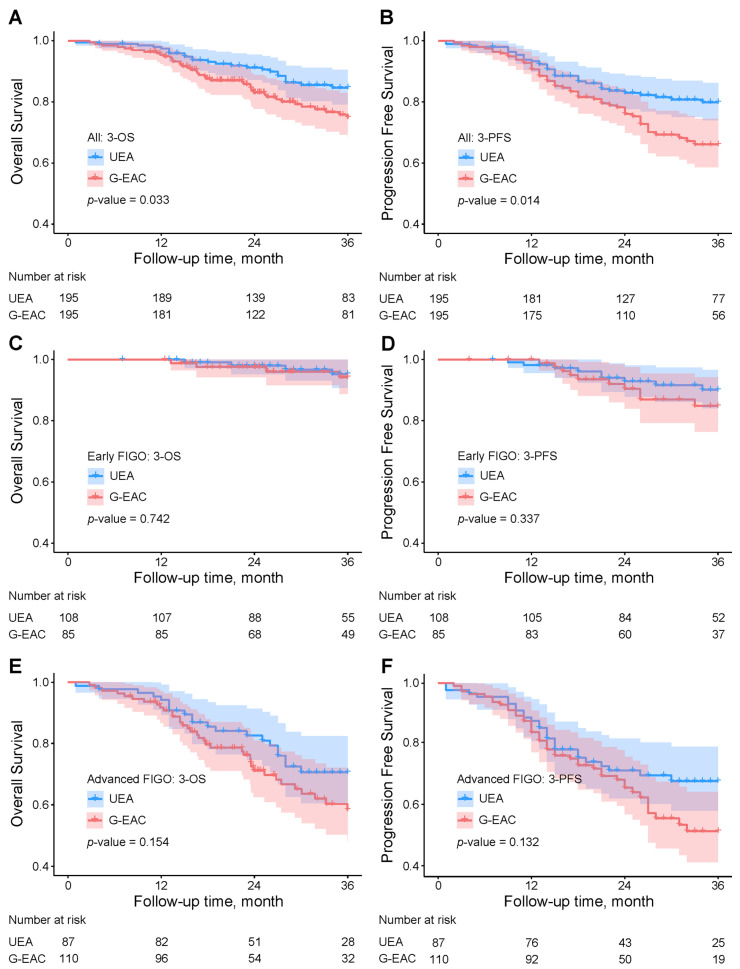
Kaplan–Meier curves for three-year overall survival and progression-free survival in usual-type and gastric-type endocervical adenocarcinoma cohorts. (**A**,**B**) All 195 matched patients per group. (**C**,**D**) Early-stage patients (FIGO < IIB; 108 usual-type and 85 gastric-type). (**E**,**F**) Advanced-stage patients (FIGO ≥ IIB; 87 usual-type and 110 gastric-type). UEA, usual-type endocervical adenocarcinoma; G-EAC, gastric-type endocervical adenocarcinoma; OS, overall survival; PFS, progression-free survival.

Patients with G-EAC had lower three-year overall survival (74.9% vs. 84.6%, *p* = 0.033) and progression-free survival (66.1% vs. 79.8%, *p* = 0.014) than those with usual endocervical adenocarcinoma (Figure 2A,B; Table S3). When stratified by FIGO stage, survival was similar between subtypes in early-stage disease (FIGO < IIB; Figure 2C,D; Table S3). In advanced-stage patients (FIGO ≥ IIB), G-EAC showed reduced overall survival (58.4% vs. 70.6%, *p* = 0.154) and progression-free survival (50.1% vs. 66.9%, *p* = 0.132), though differences were not statistically significant (Figure 2E,F; Table S3).

Univariate analysis showed that most pathological features were associated with poor prognosis in both groups (Table S4). In G-EAC, parametrial invasion and lymph node metastasis independently predicted mortality and recurrence (Table 2). In usual-type endocervical adenocarcinoma, ovarian metastasis, parametrial invasion, and lymph node involvement predicted mortality, while deep stromal invasion, positive margins, and lymph node metastasis predicted recurrence (Table 2).

Cox regression indicated that adjuvant therapy was associated with worse outcomes overall, likely reflecting more advanced disease, but provided survival benefits in high-risk subgroups. Patients with parametrial invasion or advanced FIGO stage experienced significantly improved overall survival and moderately better progression-free survival, while benefits were less pronounced in those with pelvic lymph node metastasis (Figure S1).

Among 351 patients receiving defined adjuvant regimens, Cox analysis showed no overall survival differences by therapy type. In interaction analysis, patients with G-EAC had significantly worse overall survival and progression-free survival than those with usual endocervical adenocarcinoma when treated with radiotherapy or chemotherapy alone (HRs: 5.88 and 8.37) (Figure S2). Even with combined chemoradiotherapy, G-EAC remained associated with poorer outcomes (overall survival HR 2.61 vs. 1.63; progression-free survival HR 3.91 vs. 2.66), indicating reduced treatment responsiveness (Figure S2).

**Figure 3:**
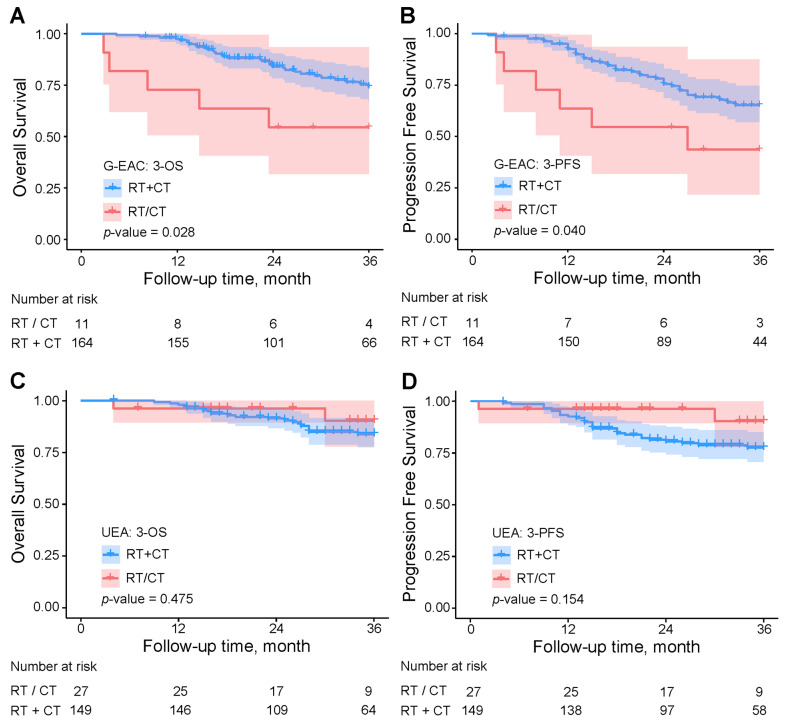
Kaplan–Meier curves of three-year overall and progression-free survival by adjuvant therapy type. (**A**,**B**) First, 164 G-EAC patients receiving combined therapy (RT + CT) and 11 receiving single-modality therapy (RT/CT), while (**C**,**D**) show 149 usual-type endocervical adenocarcinoma patients with combined therapy and 27 with single-modality therapy. UEA, usual-type endocervical adenocarcinoma; G-EAC, gastric-type endocervical adenocarcinoma; OS, overall survival; PFS, progression-free survival; RT: radiotherapy; CT: chemotherapy.

Kaplan–Meier curves showed that combined therapy significantly improved 3-year overall survival and progression-free survival in G-EAC compared with single-modality treatment (overall survival 74.3% vs. 54.5%, *p* = 0.028; progression-free survival 65.2% vs. 43.6%, *p* = 0.040) (Figure 3A,B). In usual endocervical adenocarcinoma, combined therapy tended to yield worse outcomes, though differences were not significant (Figure 3C,D).

## 4. Discussion

### 4.1. Main Findings

G-EAC is an aggressive cervical cancer. After propensity score matching, three-year overall and progression-free survival were significantly lower than in usual-type endocervical adenocarcinoma, indicating its poor prognosis is independent of age, surgical approach, FIGO stage, or adjuvant therapy.

### 4.2. Strengths and Weaknesses

To our knowledge, this is the largest study of G-EAC to date, offering valuable insights into its clinical, pathological, and prognostic characteristics. However, the study has several limitations. Despite its large sample size, the follow-up period is relatively short, preventing the estimation of long-term survival and recurrence. Additionally, as a single-center retrospective study conducted at a leading obstetrics and gynecology hospital in China, the patient population may be subject to selection bias. Patients at this hospital generally have higher socioeconomic status and educational levels, which could limit the generalizability of the findings. Furthermore, this study lacks an investigation into salvage therapies for recurrent cases. Future research should involve rigorously designed prospective cohort or randomized controlled trials to identify optimal treatment regimens for G-EAC. As noted by the 2013 International Rare Tumor Initiative [18], prospective studies on rare cancers should involve multi-center and international collaborations. In the context of rare histological subtypes like G-EAC, the reliance on single-center cohorts—despite being based on high-volume tertiary hospitals—presents inherent challenges in achieving statistical power and global generalizability. Therefore, the establishment and expansion of prospective international databases are paramount.

The history of G-EAC diagnostic criteria further underscores the necessity for such collaboration. The concept was first proposed by Japanese scholars in 2007, and it was previously known as minimal deviation adenocarcinoma or malignant adenoma. It was not until the 2014 WHO Classification (4th edition) that G-EAC began to be relatively standardized, and subsequently, the International Endocervical Adenocarcinoma Criteria and Classification (IECC) project in 2018 established the pivotal framework for HPV-associated and non-associated categories—a classification officially adopted by the 2020 WHO Classification (5th edition).

However, significant barriers to global collaboration persist. In many regions, the low incidence of G-EAC and a lack of diagnostic expertise in smaller centers lead to missed or delayed identification. These diagnostic hurdles, coupled with historically inconsistent nomenclature, have hindered the integration of multi-center data. Nevertheless, international synergy remains essential. Such collaborative platforms facilitate the aggregation of standardized clinicopathological data across diverse ethnic populations. This synergy is crucial for refining diagnostic criteria, validating prognostic biomarkers, and conducting robust clinical trials for targeted therapies that are otherwise unfeasible in a single-institution setting. Future research should prioritize the integration of localized cohorts into these international registries to foster a more comprehensive understanding of G-EAC and to develop unified, evidence-based management protocols on a global scale.

### 4.3. Interpretation

Previous studies have reported poor prognosis for G-EAC, though most involved small cohorts [8,12,13,14,19,20,21,22,23,24,25,26,27] (Table S5). Kojima et al. [8] first described 16 cases, showing lower five-year disease-free survival than non-gastric mucinous adenocarcinoma (30% vs. 77%, *p* < 0.005) and higher recurrence risk. Karamurzin et al. [12], analyzing 40 cases over 20 years, found five- and ten-year disease-specific survival significantly lower than in usual-type endocervical adenocarcinoma (5-year/10-year: 42% vs. 91%, *p* < 0.0001), with consistent results among stage I patients (62% vs. 96%, *p* = 0.0003), highlighting the aggressive nature of this subtype despite limited sample size.

In our study, stratification by FIGO stage showed a trend toward worse prognosis for G-EAC compared with usual-type endocervical adenocarcinoma, though not statistically significant, likely due to limited follow-up and sample size. The difference was more pronounced in advanced-stage patients, possibly reflecting reduced responsiveness to postoperative adjuvant therapy.

A secondary analysis of 102 G-EAC cases from a multicenter retrospective study stratified patients into high-, medium-, and low-risk groups. In the medium-risk group (*n* = 37), radiotherapy alone or no adjuvant therapy showed better prognostic trends than chemotherapy or concurrent chemoradiotherapy. In the high-risk group (*n* = 48), radiotherapy also trended toward better outcomes, though this was not statistically significant due to the small sample size [14].

Kojima et al. [13] reanalyzed a phase II trial (SGSG005) on neoadjuvant chemotherapy for non-squamous cervical cancer, including 13 gastric-type and 20 usual-type endocervical adenocarcinoma cases. The response rate to neoadjuvant chemotherapy (docetaxel and carboplatin) was significantly lower in gastric-type cases than in usual-type (46.2% vs. 85.0%, *p* = 0.048).

Due to the large population of China and the relative inaccessibility of radiotherapy resources, many patients requiring postoperative adjuvant therapy face long waiting times for radiation. To bridge this gap, clinicians typically administer one to two cycles of chemotherapy (paclitaxel and carboplatin) before radiation, making combined radiotherapy and chemotherapy the main postoperative regimen in our cohort.

In 175 G-EAC patients receiving adjuvant therapy, combined radiotherapy and chemotherapy significantly improved 3-year progression-free survival and overall survival compared to either treatment alone (χ^2^ = 4.23/4.86, *p* = 0.0397/0.0275). Cox regression showed that, compared to usual-type endocervical adenocarcinoma, gastric-type patients receiving radiotherapy or chemotherapy alone had markedly higher risks of death and recurrence (3-year overall survival/progression-free survival HR: 5.88/8.37). Even with combined therapy, gastric-type patients had higher risks than usual-type (3-year overall survival HR: 2.61 vs. 1.63; 3-year progression-free survival HR: 3.91 vs. 2.66), indicating reduced responsiveness, though combined therapy may partially mitigate risk. Further studies are needed to optimize treatment for this subtype.

A study evaluating HER2 expression in 58 G-EAC cases [28]. The results show that HER2 overexpression and amplification were observed in 17.2% and 15.5% of patients, respectively, with 90% concordance between immunohistochemistry and fluorescence in situ hybridization, suggesting potential for targeted therapy such as trastuzumab. A Japanese study of 13 cases identified 74 genomic alterations across 42 genes [29], most frequently in TP53, ATRX, CDKN2A, KRAS, APC, and STK11, highlighting genomic changes that affect signaling pathways and cell cycle regulation. Molecular profiling may guide precision medicine approaches for this aggressive subtype.

A Chinese cohort study using whole-exome sequencing of tumors and adjacent tissues from 25 G-EAC patients revealed frequent mutations in TP53 (32%), CDKN2A (20%), STK11 (20%), BRCA2 (12%), SMAD4 (12%), and ERBB2 (12%), as well as previously unreported recurrent mutations in PBRM1 (12%), FRMPD4 (12%), and NOP2 (8%) [21]. Somatic copy number gains of APOBEC3B were associated with better prognosis, and positive APOBEC3B staining may serve as a biomarker for favorable outcomes. These findings highlight the potential for targeted molecular therapies guided by genetic profiling.

G-EAC is rare, especially in Western populations, but accounts for 20–25% of cervical adenocarcinomas in Japan [30]. In our cohort, it represented 18.9% of endocervical adenocarcinomas, consistent with other Chinese studies (15.9–18.2%) [20]. To our knowledge, this is the largest study on G-EAC to date, providing valuable clinical, pathological, and prognostic insights. G-EAC patients were diagnosed at a significantly older age than usual endocervical adenocarcinoma patients, consistent with a recent Chinese study showing younger age in HPV-associated versus HPV-independent cases [31]. Additionally, we found that preoperative serum CA199 was significantly higher in G-EAC than in usual endocervical adenocarcinoma, consistent with results from Shi et al. [32] and Chen et al. [24], supporting its potential use for diagnosis and screening. Furthermore, our study found that G-EAC patients had a higher rate of ovarian metastasis (15%) than usual-type. This finding corroborates previous research by Ehmann et al. [22]. Tian et al. [19] reported that G-EAC is more likely to involve lymph node metastasis, deep stromal invasion, uterine corpus invasion, and recurrence compared to usual-type. Similarly, our study suggests that G-EAC tumors are larger, invade deeper into the cervix, and more frequently involve the parametrium and vagina than usual endocervical adenocarcinoma, reflecting more aggressive behavior.

Since G-EAC is HPV-independent and typically occurs in the upper segment of the cervix, conventional cervical screening often fails to detect it, leading to diagnoses at advanced stages. Karamurzin et al. [12] reported that 59% of G-EAC patients were diagnosed at FIGO stages II-IV, versus 41% at stage I. Consistently, in our study, 55.9% of G-EAC patients were diagnosed at FIGO stage IIB or higher, compared to 12.4% of usual endocervical adenocarcinoma patients. These findings highlight the need for early screening to improve G-EAC prognosis.

G-EAC is a rare cervical cancer subtype with markedly worse prognosis than usual endocervical adenocarcinoma. Postoperative combined radiotherapy and chemotherapy lower recurrence and mortality risks, but G-EAC remains less responsive to adjuvant chemoradiotherapy than usual endocervical adenocarcinoma.

Further research is required to investigate the mechanisms underlying the development of G-EAC, its genetic drivers, and potential targeted therapies. Emphasis should be placed on precision treatments guided by genetic profiles, alongside early screening to improve prognosis through timely intervention.

## 5. Conclusions

G-EAC is a rare, aggressive cervical cancer subtype with a markedly poorer prognosis than usual endocervical adenocarcinoma. Parametrial invasion and pelvic lymph node metastasis independently increase the risks of recurrence and death. Combined postoperative radiotherapy and chemotherapy improve outcomes compared to single-modality treatment, but remain less responsive than usual endocervical adenocarcinoma. Further research is needed to develop more effective therapies for this rare subtype.

## Figures and Tables

**Table 1:** Comparison of tumor markers, pathological, and outcome characteristics after propensity score matching.

	G-EAC (*n* = 195)	UEA (*n* = 195)	*p*-Value
Tumor markers			
CA199	44.26 [11.54, 423.43]	9.73 [4.76, 25.31]	<0.001
CA125	17.80 [11.96, 36.38]	19.51 [13.04, 31.97]	0.327
Pathological			
Ovarian metastasis			
No	159 (81.5%)	156 (80.0%)	0.003
Yes	28 (15.0%)	8 (4.9%)	
Max diameter > 4 cm			
No	114 (58.5%)	147 (75.4%)	0.002
Yes	75 (39.7%)	47 (24.2%)	
Invasion Depth of Cervical Stroma			
Superficial 1/3	17 (8.7%)	34 (17.4%)	<0.001
Middle 1/3	13 (6.7%)	38 (19.5%)	
Deep 1/3	165 (84.6%)	123 (63.1%)	
Parametrium invasion			
No	143 (73.3%)	167 (85.6%)	0.001
Unilateral	30 (15.4%)	23 (11.8%)	
Bilateral	22 (11.3%)	5 (2.6%)	
Positive surgical margins			
No	162 (83.1%)	187 (95.9%)	<0.001
Yes	33 (16.9%)	8 (4.1%)	
Vaginal invasion			
No	108 (55.4%)	144 (73.8%)	<0.001
Yes	87 (44.6%)	51 (26.2%)	
Lymph node metastasis			
No	104 (53.3%)	114 (58.5%)	0.504
Para-aortic	14 (7.2%)	10 (5.1%)	
Pelvic	77 (39.5%)	71 (36.4%)	
Lymphovascular Invasion			
No	36 (18.5%)	51 (26.2%)	0.063
Yes	152 (80.9%)	132 (72.1%)	
Outcome			
Recurrence			
No	142 (72.8%)	160 (82.1%)	0.039
Yes	53 (27.2%)	35 (17.9%)	
Death			
No	155 (79.5%)	170 (87.2%)	0.057
Yes	40 (20.5%)	25 (12.8%)	

Tumor markers include CA199 and CA125, and others are post-operative pathological characteristics. Comparison was conducted in 390 patients after 1:1 propensity score matching, which balanced demographic (age, ethnicity, marital status), clinical (year of diagnosis, BMI, Karnofsky performance status, menopausal status, fertility status, FIGO stage), treatment (surgical approach, postoperative adjuvant therapy) covariates. Continuous variables are described as mean ± standard deviation (SD) or median [interquartile range]. Categorial variables are described as frequency (percentage). *p* values were obtained using *t* test of Wilcoxon rank-sum test for continuous variables and Chi-square test for categorical variables. UEA, usual-type endocervical adenocarcinoma; G-EAC, gastric-type endocervical adenocarcinoma.

**Table 2:** Results of Cox Multivariate Analysis.

**Pathological Characters**	**G-EAC: 3-Year OS**			**UEA: 3-Year OS**		
	**HR**	**95% CI**	***p*-Value**	**HR**	**95% CI**	***p*-Value**
Ovarian metastasis (yes vs. no)	2.03	[0.94, 4.35]	0.070	2.43	[1.3, 4.55]	**0.006**
Max diameter > 4 cm (yes vs. no)	-	-	-	0.96	[0.56, 1.66]	0.882
Invasion deep 1/3 of Cervical Stroma (yes vs. no)	1.60	[0.18, 14.42]	0.676	2.31	[0.90, 5.95]	0.083
Parametrium invasion (yes vs. no)	3.19	[1.46, 7.00]	**0.004**	2.06	[1.13, 3.74]	**0.018**
Positive surgical margins (yes vs. no)	1.32	[0.62, 2.84]	0.475	1.87	[0.97, 3.59]	0.060
Vaginal invasion (yes vs. no)	0.96	[0.41, 2.27]	0.927	1.35	[0.72, 2.54]	0.350
Lymph node metastasis						
Para-aortic (vs. no)	1.86	[0.49, 7.02]	0.361	4.58	[1.78, 11.77]	**0.002**
Pelvic (vs. no)	2.64	[1.24, 5.65]	**0.012**	3.44	[1.83, 6.44]	**<0.001**
Lymphovascular Invasion (yes vs. no)	1.28	[0.34, 4.88]	0.717	2.25	[0.89, 5.71]	0.088
**Pathological Characters**	**G-EAC: 3-Year PFS**			**UEA: 3-Year PFS**		
	**HR**	**95% CI**	***p*-Value**	**HR**	**95% CI**	***p*-Value**
Ovarian metastasis (yes vs. no)	1.70	[0.83, 3.50]	0.147	1.59	[0.88, 2.90]	0.127
Max diameter > 4 cm (yes vs. no)	1.03	[0.55, 1.94]	0.925	1.18	[0.74, 1.89]	0.488
Invasion deep 1/3 of Cervical Stroma (yes vs. no)	2.10	[0.47, 9.50]	0.334	4.87	[2.07, 11.49]	**<0.001**
Parametrium invasion (yes vs. no)	2.54	[1.26, 5.11]	**0.009**	1.75	[1.03, 2.99]	**0.039**
Positive surgical margins (yes vs. no)	1.90	[0.96, 3.76]	0.067	1.88	[1.06, 3.34]	**0.031**
Vaginal invasion (yes vs. no)	1.05	[0.50, 2.21]	0.891	1.40	[0.83, 2.36]	0.202
Lymph node metastasis						
Para-aortic (vs. no)	1.17	[0.31, 4.35]	0.820	2.95	[1.26, 6.92]	**0.013**
Pelvic (vs. no)	2.26	[1.18, 4.31]	**0.013**	2.85	[1.71, 4.77]	**<0.001**
Lymphovascular Invasion (yes vs. no)	-	-	-	1.25	[0.64, 2.42]	0.511

Only significant pathological features (*p* < 0.05) were included in cox multivariate analysis. “-” represents this feature was not included. Bold font represents *p* value < 0.05 and corresponding feature is an independent risk factor. OS, overall survival; PFS, progression-free survival; HR, hazard ratio; CI, confidence interval.

## Data Availability

The data supporting this study’s findings are available from the corresponding author upon reasonable request. Due to privacy and ethical considerations, these data are not publicly accessible.

## Supplementary Materials

The following supporting information can be downloaded at: https://www.mdpi.com/article/10.3390/cancers18071168/s1, Figure S1. Forest plot of the main and interaction effects of postoperative adjuvant therapy and high-risk factors; Figure S2. Forest plot of the main and interaction effects of postoperative adjuvant therapy by pathological type; Table S1. Comparison of Demographic, Clinical, Treatment, Tumor Markers, Pathological, and Outcome Characteristics; Table S2. Comparison of Covariates after matching; Table S3. Comparison of 3-year OS and 3-year PFS between G-EAC and UEA cohorts; Table S4. Results of the Log-rank Univariate Analysis; Table S5. Summary of Chinese and International Studies on G-EAC (1990–2024). References [8,12,13,14,19,20,21,22,23,24,25,26,27] are cited in the Supplementary Materials.

## Abbreviations

The following abbreviations are used in this manuscript:

G-EAC	Gastric-type endocervical adenocarcinoma
UEA	Usual endocervical adenocarcinoma
PSM	Propensity score matching
PFS	progression-free survival
OS	overall survival
HPV	human papillomavirus
IECC	International Endocervical Adenocarcinoma Criteria and Classification
KPS	Karnofsky Performance Status
LVSI	lymphovascular space invasion
SD	standard deviation
SMD	Standardized mean difference
