# Supplementary material for: Clinicopathological and Prognostic Characteristics of Gastric-Type Endocervical Adenocarcinoma: A Nested Case–Control Study
Source: Cancers (Basel). 2026 Apr 4;18(7):1168. doi: 10.3390/cancers18071168 (PMC13072354; doi:10.3390/cancers18071168)
Supplement: Supplementary file 1 [file cancers-18-01168-s001.zip › Supplementary Figures.pdf]

Supplementary Figures

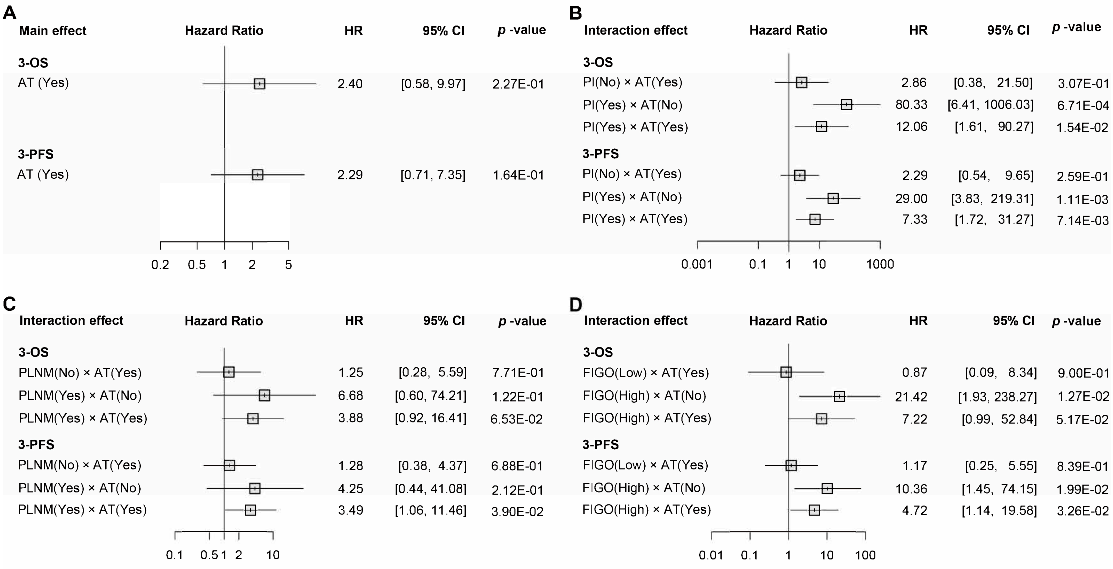

Figure S1. Forest plot of the main and interaction effects of postoperative adjuvant therapy and high-risk factors. A shows the main effect of postoperative adjuvant therapy. B, C, and D display the interaction effects of postoperative adjuvant therapy with parametrium invasion, pelvic lymph node metastasis, and advanced FIGO stages, respectively. AT, Adjuvant therapy; PI, Parametrium invasion; PLNM, Pelvic lymph node metastasis; CI, Confidence interval; OS, overall survival; PFS, progression free survival.

Effect	HR	95% CI	*p* -value
**3-OS**			
Main effect (RT+CT)	0.87	[0.40, 1.93]	7.39E-01
Interaction effect			
UEA × RT+CT	1.63	[0.38, 6.99]	5.08E-01
G-EAC × RT/CT	5.88	[1.12, 30.92]	3.66E-02
G-EAC × RT+CT	2.61	[0.63, 10.90]	1.88E-01
**3-PFS**			
Main effect (RT+CT)	1.12	[0.54, 2.32]	7.68E-01
Interaction effect			
UEA × RT+CT	2.66	[0.63, 11.12]	1.81E-01
G-EAC × RT/CT	8.37	[1.67, 42.00]	9.86E-03
G-EAC × RT+CT	3.91	[0.95, 16.17]	5.94E-02

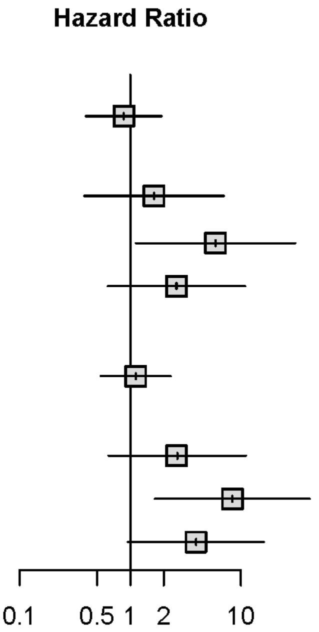

Figure S2. Forest plot of the main and interaction effects of postoperative adjuvant therapy by pathological type. For 351 patients with defined adjuvant therapy (176 usual-type and 175 gastric-type endocervical adenocarcinoma), Cox regression assessed its impact on prognosis and interaction with pathology. As we did not perform Cox regression across the full matched cohort (i.e., no between-group comparison was modeled using the matched pairs), the matched nature was taken into accounted for in our Cox models. UEA, usual-type endocervical adenocarcinoma; G-EAC, gastric-type endocervical adenocarcinoma; OS, overall survival; PFS, progression free survival.
