# Supplementary material for: Clinicopathological and Prognostic Characteristics of Gastric-Type Endocervical Adenocarcinoma: A Nested Case–Control Study
Source: Cancers (Basel). 2026 Apr 4;18(7):1168. doi: 10.3390/cancers18071168 (PMC13072354; doi:10.3390/cancers18071168)
Supplement: Supplementary file 1 [file cancers-18-01168-s001.zip › Supplementary Tables.pdf]

Supplementary Tables

Table S1: Comparison of Demographic, Clinical, Treatment, Tumor Markers, Pathological, and Outcome Characteristics.

	G-EAC（n=195）	UEA（n=765）	p-value
Demographic			
Age	50.29±11.80	46.76±9.54	<0.001
Ethnic			
Han	194（99.5%）	759（99.2%）	>0.999
Minorities	1（0.5%）	6（0.8%）	
Marital status			
Single	6（3.1%）	10（1.3%）	0.372
Married	178（91.3%）	711（92.9%）	
Divorced or Widowed	10（5.1%）	38（5.0%）	
Unknown	1（0.5%）	6（0.8%）	
Clinical			
Diagnosis year			
2018	31（15.9%）	102（13.3%）	0.152
2019	28（14.4%）	124（16.2%）	
2020	31（15.9%）	113（14.8%）	
2021	34（17.4%）	147（19.2%）	
2022	40（20.5%）	110（14.4%）	
2023	31（15.9%）	169（22.1%）	
BMI			
<18.5	10（5.1%）	32（4.2%）	0.812
18.5~23.9	110（56.4%）	459（60.0%）	
24.0~27.9	61（31.3%）	223（29.2%）	
>28.0	14（7.2%）	51（6.7%）	
KPS			
90	20（10.3%）	46（6.0%）	0.053
100	175（89.7%）	719（94.0%）	
Menopause			
No	107（54.9%）	533（69.7%）	<0.001
Yes	88（45.1%）	232（30.3%）	
Parous			
No	14（6.7%）	38（5.0%）	0.444
Yes	181（93.3%）	727（95.0%）	
FIGO stage			
IA1、IA2	3（1.5%）	128（16.7%）	<0.001
IB1、IB2、IIA1	69（35.4%）	498（65.1%）	
IB3、IIA2	13（6.7%）	43（5.6%）	
IIB	14（7.2%）	6（0.8%）	
IIIA、IIIB、IIIC1、IIIC2	90（46.2%）	87（11.4%）	
IVB	6（3.1%）	3（0.4%）	
Treatment			
Surgical approach			
laparoscopy/robot	166（85.1%）	722（94.4%）	<0.001
laparotomy	29（14.9%）	43（5.6%）	
Adjuvant therapy			
No	17（8.7%）	428（55.9%）	<0.001
Yes	175（89.7%）	314（41.0%）	
Recommend	3（1.5%）	23（3.0%）	

Tumor markers			
CA199	44.26 [11.54, 423.43]	8.74 [3.90, 17.98]	<0.001
CA125	17.80 [11.96, 36.38]	15.39 [11.23, 23.67]	0.007
Pathological			
Ovarian metastasis			
No	159（81.5%）	514 (67.2%)	<0.001
Yes	28（15.0%）	13 (1.7%)	
Max diameter > 4 cm			
No	114（58.5%）	684 (89.4%)	<0.001
Yes	75（39.7%）	80 (10.5%)	
Invasion Depth of Cervical Stroma			
Superficial 1/3	17（8.7%）	412 (53.9%)	<0.001
Middle 1/3	13（6.7%）	147 (19.2%)	
Deep 1/3	165（84.6%）	206 (26.9%)	
Parametrium invasion			
No	143（73.3%）	736 (96.2%)	<0.001
Unilateral	30（15.4%）	24 (3.1%)	
Bilateral	22（11.3%）	5 (0.7%)	
Positive surgical margins			
No	162（83.1%）	757 (99.0%)	<0.001
Yes	33（16.9%）	8 (1.0%)	
Vaginal invasion			
No	108（55.4%）	680 (88.9%)	<0.001
Yes	87（44.6%）	85 (11.1%)	
Lymph node metastasis			
No	104（53.3%）	679 (88.8%)	<0.001
Para-aortic	14（7.2%）	10 (1.3%)	
Pelvic	77（39.5%）	76 (9.9%)	
Lymphovascular Invasion			
No	36（18.5%）	519 (67.8%)	<0.001
Yes	152（80.9%）	234 (30.6%)	
Outcome			
Recurrence			
No	142（72.8%）	715 (93.5%)	<0.001
Yes	53（27.2%）	50 (6.5%)	
Death			
No	155（79.5%）	733 (95.8%)	<0.001
Yes	40（20.5%）	32 (4.2%)	

Continuous variables are described as mean ± standard deviation (SD) or median [interquartile range]. Categorial variables are described as frequency (percentage). *p* -Values were obtained using t test of Wilcoxon rank-sum test for continuous variables and Chi-square test for categorical variables. KPS, Karnofsky performance status; UEA, usual-type endocervical adenocarcinoma; G-EAC, gastric-type endocervical adenocarcinoma

Table S2. Comparison of Covariates after matching.

	G-EAC（n=195）	UEA（n=765）	p-value	SMD
Demographic				
Age	50.29±11.80	50.04±10.66	0.825	0.022
Ethnic				

Han	194（99.5%）	194（100.0%）	>0.999	<0.001
Minorities	1（0.5%）	1（0.5%）		
Marital status				
Single	6（3.1%）	3（1.5%）	0.679	0.125
Married	178（91.3%）	178（91.3%）		
Divorced or widowed	10（5.1%）	12（6.2%）		
Unknown	1（0.5%）	2（1.0%）		
Clinical				
Diagnosis year				
2018	31（15.9%）	33（16.9%）	0.928	0.118
2019	28（14.4%）	34（17.4%）		
2020	31（15.9%）	30（15.4%）		
2021	34（17.4%）	35（17.9%）		
2022	40（20.5%）	33（16.9%）		
2023	31（15.9%）	30（15.4%）		
BMI				
<18.5	10（5.1%）	6（3.1%）	0.701	0.121
18.5~23.9	110（56.4%）	107（54.9%）		
24.0~27.9	61（31.3%）	68（34.9%）		
>28.0	14（7.2%）	14（7.2%）		
KPS				
90	20（10.3%）	14（7.2%）	0.369	0.109
100	175（89.7%）	181（92.8%）		
Menopause				
No	107（54.9%）	107（54.9%）	>0.999	<0.001
Yes	88（45.1%）	88（45.1%）		
Parous				
No	13（6.7%）	10（5.1%）	0.667	0.065
Yes	182（93.3%）	185（94.9%）		
FIGO stage (fine-grained)				
IA1、IA2	3（1.5%）	6（3.1%）	0.105	0.309
IB1、IB2、IIA1	69（35.4%）	85（43.6%）		
IB3、IIA2	13（6.7%）	17（8.7%）		
IIB	14（7.2%）	6（3.1%）		
IIIA、IIIB、IIIC1、IIIC2	90（46.2%）	79（40.5%）		
IVB	6（3.1%）	2（1.0%）		
FIGO stage (coarse-grained)				
I, II	99 (50.8%)	114 (58.5%)	0.760	0.041
III, IV	96 (49.2%)	81 (41.5%)		
Treatment				
Surgical approach				
laparoscopy/robot	166（85.1%）	174（89.2%）	0.289	0.123
laparotomy	29（14.9%）	21（10.8%）		
Adjuvant therapy				
No	17（8.7%）	14（7.2%）	0.854	0.057
Yes	175（89.7%）	178（91.3%）		
Recommend#	3（1.5%）	3（1.5%）		

Continuous variables are described as mean ± standard deviation (SD) or median [interquartile range]. Categorial variables are described as frequency (percentage). *p* -Values were obtained using t test of Wilcoxon rank-sum test for continuous variables and Chi-square test for categorical variables. SMD, standard mean difference; KPS, Karnofsky performance status; UEA, usual-type endocervical adenocarcinoma; G-EAC, gastric-type endocervical adenocarcinoma.

"Recommend" for adjuvant therapy indicates a recommendation for postoperative adjuvant therapy, but due to loss to follow-up, it is not known whether the patient completed the adjuvant treatment.

Table S3. Comparison of 3-year OS and 3-year PFS between G-EAC and UEA cohorts.

	N	3-Year OS			3-Year PFS		
		OS	95% CI	*p* -Values	PFS	95% CI	*p* -Values
All stages							
UEA	195	84.6%	[79.2%, 90.5%]	0.033	79.8%	[74.0%, 86.2%]	0.014
G-EAC	195	74.9%	[68.2%, 82.2%]		66.1%	[58.6%, 74.4%]	
FIGO stages < IIB							
UEA	108	95.2%	[90.7%, 100%]	0.742	90.1%	[84.0%, 96.6%]	0.337
G-EAC	85	94.2%	[88.7%, 100%]		84.8%	[76.3%, 94.2%]	
FIGO stages ≥ IIB							
UEA	87	70.6%	[60.5%, 82.4%]	0.154	66.9%	[57.1%, 78.5%]	0.132
G-EAC	110	58.4%	[48.4%, 70.6%]		50.1%	[39.7%, 63.3%]	

UEA, usual-type endocervical adenocarcinoma; G-EAC, gastric-type endocervical adenocarcinoma; OS, overall survival; PFS, progression free survival; HR, hazard ratio; CI, confidence interval.

Table S4. Results of the Log-rank Univariate Analysis.

Pathological characters	G-EAC: 3-Year OS			UEA: 3-Year OS		
	HR	95% CI	*p* -Values	HR	95% CI	*p* -Values
Ovarian metastasis (yes vs. no)	4.11	[2.07, 8.17]	<0.001	7.87	[4.46, 13.87]	<0.001
Max diameter > 4 cm (yes vs. no)	1.74	[0.93, 3.27]	**0.084**	4.47	[2.79, 7.17]	<0.001
Invasion deep 1/3 of Cervical Stroma (yes vs. no)	8.15	[1.12, 59.31]	0.038	12.61	[6.27, 25.36]	<0.001
Parametrium invasion (yes vs. no)	5.34	[2.84, 10.03]	<0.001	12.34	[7.71, 19.78]	<0.001
Positive surgical margins (yes vs. no)	2.38	[1.19, 4.77]	0.015	7.21	[4.01, 12.94]	<0.001
Vaginal invasion (yes vs. no)	3.22	[1.65, 6.25]	<0.001	7.89	[4.93, 12.62]	<0.001
Lymph node metastasis						
Para-aortic (vs. no)	2.64	[0.73, 9.48]	0.137	19.94	[9.10, 43.69]	<0.001
Pelvic (vs. no)	4.05	[2.00, 8.22]	<0.001	12.78	[7.56, 21.61]	<0.001
Lymphovascular Invasion (yes vs. no)	3.44	[1.06, 11.17]	0.040	14.88	[6.82, 32.46]	<0.001

Pathological characters	G-EAC: 3-Year PFS			UEA: 3-Year PFS		
	HR	95% CI	*p*-value	HR	95% CI	*p*-value
Ovarian metastasis (yes vs. no)	2.64	[1.38, 5.07]	0.004	5.09	[2.95, 8.76]	<0.001
Max diameter > 4 cm (yes vs. no)	2.00	[1.16, 3.47]	0.013	5.17	[3.49, 7.66]	<0.001
Invasion deep 1/3 of Cervical Stroma (yes vs. no)	3.87	[1.21, 12.43]	0.023	11.31	[6.53, 19.58]	<0.001
Parametrium invasion (yes vs. no)	3.85	[2.23, 6.65]	<0.001	9.22	[6.11, 13.91]	<0.001
Positive surgical margins (yes vs. no)	2.32	[1.26, 4.29]	0.007	6.75	[4.04, 11.28]	<0.001
Vaginal invasion (yes vs. no)	2.52	[1.44, 4.40]	0.001	6.76	[4.57, 9.98]	<0.001
Lymph node metastasis						
Para-aortic (vs. no)	1.59	[0.47, 5.40]	0.458	12.65	[6.30, 25.43]	<0.001
Pelvic (vs. no)	3.15	[1.76, 5.63]	<0.001	9.11	[6.02, 13.79]	<0.001
Lymphovascular Invasion (yes vs. no)	1.78	[0.80, 3.95]	**0.156**	7.82	[4.69, 13.02]	<0.001

8 key pathological features were analyzed in log-rank univariate analysis. Bold font represents p -Value > 0.05 and will not be included in cox multivariate analysis. OS, overall survival; PFS, progression free survival; HR, hazard ratio; CI, confidence interval.

Table S5: Summary of Chinese and International Studies on Gastric-Type Endocervical Adenocarcinoma (1990–2024).

	Study	Organization	Research time span	Sample size (EA/G-EAC)	G-EAC survival outcome
Chinese	Xu, Pan et al. 2019 [26]	Xijing Hospital, Fourth Military Medical University	2006-2017	213/12	NA
	Chen, Niu et al. 2021 [24]	Women's Hospital, Zhejiang University	2014-2020	512 (including AIS) /38	Median PFS and OS time significantly lower than non-G-EAC
	Shi, Shao et al. 2022 [20]	Women's Hospital, Zhejiang University	2004-2019	402/77	HPVI G-EAC is a poor prognostic factor for OS/PFS (HR 1.739/1.432 compared to common HPVA)
	Liao, Xia et al. 2022 [21]	West China Hospital, Sichuan University	2017.12-2020.01	(-) /25	A median follow-up of 29 months, with 3 deaths and 11 relapses.
			2016.01-2017.12 2020.01-2021.07	(-) /33	IHC validation
	Tian, Liu et al. 2024 [19]	Multi-centers (32 centers)	2004-2018	672/34	5-year DFS: 35.6% 5-year OS: 34%
International	Kojima, Mikami et al. 2007 [8]	Hyogo Medical Center for Adults, Akashi	1991-2001	53 (EMA) /16	5-year PFS: 25% 5-year DFS: 30%

Karamurzin, Kiyokawa et al. 2015 [12]	Multi-centers (3 centers)	1997-2012	139 (UEA) /40	All stage 5-year DSS：42% 10-year DSS：31% Stage I 5-year DSS：62% 10-year DSS：62%
Kojima, Shimada et al. 2018 [13]	Multi-centers*	2007-2010	33/13	5-year PFS：38.5% 5-year OS:36.9 %
Nishio, Mikami et al. 2019 [27]	JCOG-GCSG	2000-2009	393/95	Median PFS time: 42 months
Nishio, Matsuo et al. 2022 [14]	JCOG-GCSG#	2000-2009	(-) /102	Among the high-risk groups, the radiotherapy group had a better trend in PFS and OS than the CCRT and chemotherapy groups.
Jung, Bae et al. 2020 [25]	Samsung Medical Center, Sungkyunkwan University School of Medicine	2015-2016	31/8	Median DFS time: 19 months
Radomska, Lee et al. 2021 [23]	College of Medicine, University of Saskatchewan	2000-2015	24/5	Median survival time: 22.0 months
Ehmann, Sassine et al. 2022 [22]	Memorial Sloan Kettering Cancer Center	2002-2019	(-) /43	Median PFS time: 25 months Median OS time: 44 months

* Re-analysis of the prospective Phase II clinical study SGSG005.
# Re-analysis of the above study.

Abbreviations: AIS: adenocarcinoma in situ; CCRT: concurrent chemoradiotherapy; DFS: disease-free survival; DSS: disease-specific survival; EA: endocervical adenocarcinoma; EMA: endocervical mucinous adenocarcinoma; HPVI: HPV independent endocervical adenocarcinoma; HPVA HPV associated endocervical adenocarcinoma; JCOG-GCSG: Gynecologic Cancer Study Group of the Japan Clinical Oncology Group; OS: overall survival; PFS: progression-free survival.

References

8. Kojima, A.; Mikami, Y.; Sudo, T.; Yamaguchi, S.; Kusanagi, Y.; Ito, M.; Nishimura, R. Gastric morphology and immunophenotype predict poor outcome in mucinous adenocarcinoma of the uterine cervix. *Am J Surg Pathol* **2007**, *31*, 664-672, https://doi.org/10.1097/01.pas.0000213434.91868.b0.

12. Karamurzin, Y.S.; Kiyokawa, T.; Parkash, V.; Jotwani, A.R.; Patel, P.; Pike, M.C.; Soslow, R.A.; Park, K.J. Gastric-type Endocervical Adenocarcinoma: An Aggressive Tumor With Unusual Metastatic Patterns and Poor Prognosis. *The American Journal of Surgical Pathology* **2015**, *39*, 1449-1457, https://doi.org/10.1097/PAS.0000000000000532.

13. Kojima, A.; Shimada, M.; Mikami, Y.; Nagao, S.; Takeshima, N.; Sugiyama, T.; Teramoto, N.; Kiyokawa, T.; Kigawa, J.; Nishimura, R. Chemoresistance of Gastric-Type Mucinous Carcinoma of the Uterine Cervix: A Study of the Sankai Gynecology Study Group. *International Journal of Gynecological Cancer : Official Journal of the International Gynecological Cancer Society* **2018**, *28*, 99-106, https://doi.org/10.1097/IGC.0000000000001145.

14. Nishio, S.; Matsuo, K.; Nasu, H.; Murotani, K.; Mikami, Y.; Yaegashi, N.; Satoh, T.; Okamoto, A.; Ishikawa, M.; Miyamoto, T.; et al. Analysis of postoperative adjuvant therapy in 102 patients with gastric-type mucinous carcinoma of the uterine cervix: A multi-institutional study. *Eur J Surg Oncol* **2022**, *48*, 2039-2044, https://doi.org/10.1016/j.ejso.2022.03.007.

19. Tian, X.; Liu, P.; Kang, S.; Cui, Z.; Sun, L.; Lang, J.; Chen, C. Impact of histological subtypes on clinical outcome of endocervical adenocarcinoma. *Eur J Surg Oncol* **2024**, *50*, 107977, https://doi.org/10.1016/j.ejso.2024.107977.

20. Shi, H.; Shao, Y.; Zhang, H.; Ye, L.; Xu, E.; Lu, B. Independent validation of distinct clinicopathological features and prognosis among usual-type, mucinous-type and gastric-type endocervical adenocarcinoma categorised by new WHO classification (2020). *Pathology* **2022**, *54*, 555-562, https://doi.org/10.1016/j.pathol.2021.12.301.

21. Liao, X.; Xia, X.; Su, W.; Yan, H.; Ma, Y.; Xu, L.; Luo, H.; Liu, W.; Yin, D.; Zhang, W.H.; et al. Association of recurrent APOBEC3B alterations with the prognosis of gastric-type cervical adenocarcinoma. *Gynecol Oncol* **2022**, *165*, 105-113, https://doi.org/10.1016/j.ygyno.2022.01.036.

22. Ehmann, S.; Sassine, D.; Straubhar, A.M.; Praiss, A.M.; Aghajanian, C.; Alektiar, K.M.; Broach, V.; Cadoo, K.A.; Jewell, E.L.; Boroujeni, A.M.; et al. Gastric-type adenocarcinoma of the cervix: Clinical outcomes and genomic drivers. *Gynecologic Oncology* **2022**, *167*, 458-466, https://doi.org/10.1016/j.ygyno.2022.10.003.

23. Radomska, A.; Lee, D.; Neufeld, H.; Korte, N.; Torlakovic, E.; Agrawal, A.; Chibbar, R. A retrospective study on incidence, diagnosis, and clinical outcome of gastric-type endocervical adenocarcinoma in a single institution. *Diagn Pathol* **2021**, *16*, 68, https://doi.org/10.1186/s13000-021-01129-9.

24. Chen, L.; Niu, Y.; Wan, X.; Yu, L.; Zhang, X.; Strickland, A.L.; Dong, L.; Zhou, F.; Lu, W. Clinicopathological features and outcomes in gastric-type of HPV-independent endocervical adenocarcinomas. *BMC Cancer* **2021**, *21*, 1095, https://doi.org/10.1186/s12885-021-08792-7.
25. Jung, H.; Bae, G.E.; Kim, H.M.; Kim, H.S. Clinicopathological and Molecular Differences Between Gastric-type Mucinous Carcinoma and Usual-type Endocervical Adenocarcinoma of the Uterine Cervix. *Cancer Genomics Proteomics* **2020**, *17*, 627-641, https://doi.org/10.21873/cgp.20219.
26. Xu, H.; Pan, H.; Wang, Y.; Zhang, J. Expanded study on the risk of lymphovascular space invasion and lymph node metastasis of endocervical adenocarcinoma using Pattern Classification: a single-centre analysis of 213 cases. *Pathology* **2019**, *51*, 570-578, https://doi.org/10.1016/j.pathol.2019.04.008.
27. Nishio, S.; Mikami, Y.; Tokunaga, H.; Yaegashi, N.; Satoh, T.; Saito, M.; Okamoto, A.; Kasamatsu, T.; Miyamoto, T.; Shiozawa, T.; et al. Analysis of gastric-type mucinous carcinoma of the uterine cervix - An aggressive tumor with a poor prognosis: A multi-institutional study. *Gynecol Oncol* **2019**, *153*, 13-19, https://doi.org/10.1016/j.ygyno.2019.01.022.
